# Acinetobacter baumannii as an oro-dental pathogen: a red alert!!

**DOI:** 10.1590/1678-7757-2023-0382

**Published:** 2024-05-13

**Authors:** A. S. Smiline GIRIJA

**Affiliations:** 1 Saveetha Institute of Medical and Technical Sciences Saveetha Dental College and Hospitals Department of Microbiology Chennai Tamilnadu India Saveetha Institute of Medical and Technical Sciences (SIMATS), Saveetha Dental College and Hospitals, Department of Microbiology, Chennai-600077, Tamilnadu, India.

**Keywords:** Acinetobacter baumannii, Oral health, Virulence, Biofilm, Oral cancer

## Abstract

**Objectives:**

This review highlights the existence and association of *Acinetobacter baumannii* with the oro-dental diseases, transforming this systemic pathogen into an oral pathogen. The review also hypothesizes possible reasons for the categorization of this pathogen as code blue due to its stealthy entry into the oral cavity.

**Methodology:**

Study data were retrieved from various search engines reporting specifically on the association of *A. baumannii* in dental diseases and tray set-ups. Articles were also examined regarding obtained outcomes on *A. baumannii* biofilm formation, iron acquisitions, magnitude of antimicrobial resistance, and its role in the oral cancers.

**Results:**

*A. baumannii* is associated with the oro-dental diseases and various virulence factors attribute for the establishment and progression of oro-mucosal infections. Its presence in the oral cavity is frequent in oral microbiomes, conditions of impaired host immunity, age related illnesses, and hospitalized individuals. Many sources also contribute for its prevalence in the dental health care environment and the presence of drug resistant traits is also observed. Its association with oral cancers and oral squamous cell carcinoma is also evident.

**Conclusions:**

The review calls for awareness on the emergence of *A. baumannii* in dental clinics and for the need for educational programs to monitor and control the sudden outbreaks of such virulent and resistant traits in the dental health care settings.

## Introduction

*Acinetobacter baumanii,* the so-called “most successful” nosocomial pathogen is now stealthily expanding its survival niche in the oro-dental spaces. It is a Gram-negative coccobacillus and basically an opportunistic nosocomial pathogen causing systemic illness. It is a significant pathogen exhibiting 2-10% of mortality range in patients with recalcitrant infections.^1^ In 2017, a red alert was raised by the World Health Organization (WHO) on its carbapenem resistance changing its classification as a pathogen under the critical category.^2^ *A. baumannii* is also known for its emergence and evolution as a multi-drug resistant pathogen, as well as its recent escalation as a pan-drug resistant pathogen.^3^ Oral microbiome, followed by gut microbiota, encompasses a diverse accumulation of microbial cosmos related to oro-dental diseases various complexes of bacteria attributes for the pathogenesis. In this line, in recent years, *A. baumannii* has been profiled in the oral microbiome and has been encountered in dental diseases like endodontic infections,^4^ periodontitis,^5^ and in oral and maxillofacial infections.^6^ *A. baumannii* capacity of biofilm formation and drug resistance profiles includes the bacteria under the category of oral pathogen.^7^ This review is aimed to create awareness about *A. baumannii,* evolving as an oral pathogen in the dental health care settings, and to suggest the need for the periodical monitoring of the resistant traits to avoid sudden outbreaks.

Considering the non-oral bacteria in the oral cavity, a controversy exists on its survival being transient or permanent colonizers.^8^ Moreover, confusion over the same concept prevails in literature, classifying most pathogens, such as *A. baumannii*, as extra-hospital reservoirs that affect not only immune-compromised patients, but also those who were previously healthy and became colonized or infected in health care environments, with a study finding the latter being in up to 56.5% patients.^9^ A comprehensive analysis on the oral dysbiotic state has been conducted with hospitalized COVID-19 patients, finding a significant presence of *A. baumannii* in the oral microbiota.^10^ Infection risks have also been documented in patients requiring oral surgery and, due to its significant prevalence among these cases, periodical assessments of the oral microbiota have been suggested as a preventive measure for post-surgical infections.^11^

### Virulence factors in A. baumannii

**Figure 1:**
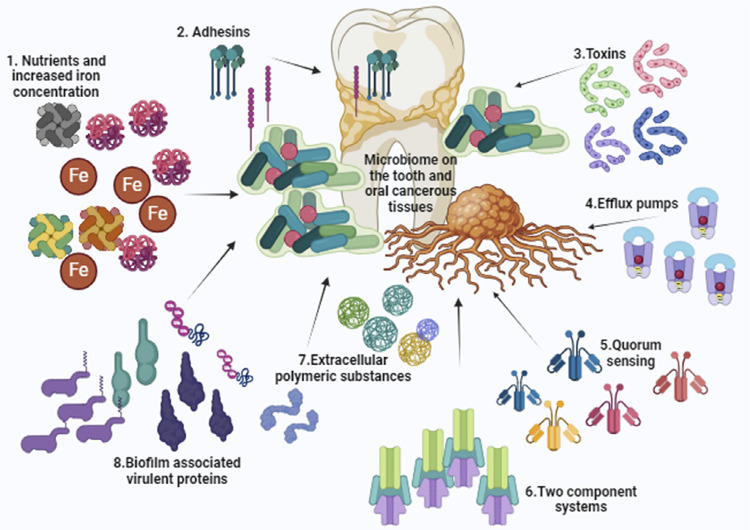
Factors contributing to the virulence of *A. baumannii* in the oral microbiota of the oro-dental infections and oral cancer. 1.The nutritional factors especially the high iron content aids in the colonization of *A. baumanii*. 2.The adhesins of bacteria play a vital role in the adherence and further multiplication on the tooth layers and oro-mucosal layers. 3.Potent toxins released by the bacteria aid in the progression of the lesions. 4.Efflux pump system helps the bacteria render resistance against antibiotics. 5.Quorum sensing factors initiate and progress the formation of biofilms. 6.Two component system of the bacteria allows bacteria to survive in the harsh environmental niche of the oral microbiome. 7.Extracellular polymeric substances aid in the adhesion, colonization, and virulence of bacteria and 8.Biofilm-associated multiple proteins attribute for the virulence and further complications of oral infections.

Evolution of *A. baumannii* as an oral pathogen in association with other oral microbiota comprises different mechanisms of virulence factors in the establishment of various oral infections. Biofilm formation is one of the major virulence mechanisms attributed by a wide variety of biofilm forming genetic determinants. Table 1 presents the biofilm-associated genes and their functions. Colonization and further progression of oral infections can be attributed to many potent virulence factors, such as adhesins, systems associated with quorum sensing, extra polymeric proteins, efflux pumps for antibiotic resistance, and the two-component systems. A mutualistic and an antagonistic interaction may prevail between *A. baumannii* and other oral bacteria but the mechanisms of these interactions and colonization are yet unclear. Amidst various discussed virulence mechanisms, the imbalance in the oral equilibrium influenced by the host immune response may be the key point for the shift of the oral bacterial microbiota with pathogens like *A. baumannii*. In contrast, *A. baumannii* does not become dominant in a well-adapted oral condition of a healthy individual.^12^ Various factors such as nutritional deprivation, light, and increased iron concentration aid in the initial attachment. Furthermore, the biofilm-associated genes present quorum sensing factors, such as alginates, to aid in the colonization and maturation of the biofilms on the tooth surface and cancerous tissues (Figure 1). Progression of oral cavity infections may be aggravated by toxins, oxygen levels in the tissues, and other poly-microbial conglomeration of the oral microbiome.^13,14^

**Table 1:** Specific genetic determinants associated with the virulence and their possible proven mechanisms in the establishment of the oro-dental infections

S.N^**o**^	Name of the genetic determinants	Functions	References
**Adhesions**			
1.	*csu* Type 1 chaperon usher pili	Surface associated proteins mediating the irreversible attachment of cells to a surface	[64]
2.	Outer membrane protein A (*ompA*)	Cell membrane integrity, drug resistance, immune-modulation, biofilm, host cell apoptosis	[65]
3.	Extracellular DNA (*eDNA*)	Biofilm and its role in cell adhesion, biofilm formation and maintenance	[66]
**QS systems**			
4.	Autoinducer luxI/luxR system	Involves acyl-homoserine lactone as an autoinducer type I	[67]
5.	l*uxS* system encoding autoinducer 2	Quorum sensing molecule	[67]
6.	AI-I acyl-homoserine lactone (AHL) QS system	Transcription of pathogenicity and biofilm-related genes	[68]
	Extra polymeric substances (EPS)		
7.	Poly- N-acetyl glucosamine (PNAG)	Major component of the biofilm matrix encoded by the *pgaABCD* locus – biofilm formation	[69]
8.	*Bap* (Biofilm associated protein)	Biofilm formation on both biotic and abiotic surfaces	[70]
**Efflux pumps**			
9.	ATP-binding cassette (ABC), resistance nodulation division (RND), small multidrug resistance (SMR), major facilitator superfamily (MFS), and multidrug and toxin-compound extrusion (MATE)	Directed towards efflux pumps' involvement in the formation of biofilm	[71]
10.	Alginate *algC*	Components of biofilm matrix	[72]
11.	Amyloidogenic proteins Curli-specific gene operon csg	Encodes for curli fibres, matrix formation, assists adhesion	[73]
**Two-component systems**			
12.	*bfmR/S*	Central regulator of biofilm formation	[74]
13.	*AdeRS*	regulate *A. baumannii* biofilm formation on plastic and mucosal tissue	[75]
14.	*FimH* Type I Fimbriae	Bacterial cell adhesion	[76]
15.	*Ptk/wzc*	Putative tyrosine kinase	[77]
16.	*kpsMII*	Group 2 capsule synthesis	[78]

### A. baumannii as an emerging oral pathogen

Emergence of *A. baumannii* as an oral pathogen is well-documented, presenting much evidence that supports its evolution as an oro-dental pathogen. Literature surveys associate its survival in the oral and mucosal layers with vital conditions favoring its colonization in the oral cavity^15,16,17^

### In oral microbiome

In recent years, *A. baumannii* has stealthily entered into the oral cavity, influenced by various host conditions and immune responses. Omics analysis of the oral samples has documented *A. baumannii* as an important part of the oral microbiome.^18,19^

The distinct substrata housing various microbial species and the permanent dental spaces aids the cumulative role for biofilm initiation by the bacterium, which is further altered by changing oral habitat, food sources, saliva flow, mastication processes, and exogenous sources of microbial entries.^20^ *A. baumannii* is now a successful pathogen in exhibiting resistance to the harsh oral stress, also becoming an inhabitant of the oro-dental spaces and is referred as “code blue” in the oral cavity.^21^ A recent study on the in-silico analysis of the *A. baumannii* genomes showed the presence of numerous virulence and resistance genes involved in its pathogenesis, documenting the bacterium as an emerging oral pathogen. It is also documented that oral cavity may be a great source for *A. baumannii* in case of poor oral hygiene and diseased conditions.^22,23^

### In impaired host immunity and diseased condition

The host health conditions also pave way for the colonization of the bacterium. It is considered as a reservoir in the oral biofilm in patients with pneumonia and other pulmonary diseases. Sub-gingival colonization of *A. baumannii* is also observed with conditions of chronic periodontitis, which was evidenced from the transcriptome analysis of the sub-gingival specimens, revealing the differential expression of different virulent factors that were responsible for attachment, progression of biofilm, and secretion of virulent proteins. Polymicrobial synergistic interactions are also responsible for its pathogenicity in promoting the refractory illnesses in the oral cavity.^24^ In this context, the formation of biofilm is known to progress via horizontal genetic transfer during the polymicrobial infection in the oral cavity into *Acinetobacter*.^25^ Moreover, the synergistic mechanisms play a vital role in enhancing the polymicrobial colonization, promoting the pathogenesis and the refractile oral infections.^26^ Host immune impairment also may enhance the colonization of the bacterium on the oro-dental spaces. The presence of polymicrobial communities in the oral cavity along with *Acinetobacter* sp. is also documented under conditions of various oro-dental diseases, systemic illnesses, and immune-deficiency conditions such as human immunodeficiency virus (HIV).^27^

Oral dysbiosis under conditions of impaired host immune response is reported from patients with diabetes, leading to chronic inflammatory burden.^28^ In hospitalized patients, *A. baumannii* is reported from the oro-pharynx and shockingly harbors integron carrying genes expressing multi-drug resistance. Comparative studies in subjects with and without periodontitis have documented *A. baumannii* as a significant pathogen causing oral infections, either alone or in association with the other red complex pathogens,^5^ and it has also been detected in the oral cavity of hospitalized patients with chronic lung disease.^29^

### Age factors

Different biological events in the host tissues are assumed to cause perturbed equilibrium inside the oral cavity and in between the complex interactions among the oral microbiomes. Especially in older adults, the incidence of the respiratory pathogens in the oral cavity is highly plausible and evidenced. In this context, presence of *A. baumannii* is documented from the oral cavity of older patients.^30^ Similarly from the older patients with ventilator associated pneumonia, *A. baumannii* was recovered from the plaque samples, revealing the same genetic traits.^31^ Moreover, a major association exists between the respiratory disease and the oral microbial communities, favoring the existence of major pathogens such as *A. baumannii* in the oral cavity. The reason behind this survival is mainly due to the homeostatic imbalance in the older population due to impaired immunity, reduced oral commensal loads and incorporation of respiratory pathogens in oral mature biofilms.^32^ The homeostatic balance may also be disturbed in young children and the situation may be worse under traumatized conditions. Orofacial cellulitis caused by *A. baumannii* have been recently reported in two pediatric cases with a fatal outcome.^33^ These literatures clearly suggest that age may be one of the key factors leading to the colonization of *A. baumannii* in the oral cavity, making it an oral pathogen.

### In intensive care units

Colonization of the oral cavity by *A. baumannii* is a major concern in intensive care units (ICUs). Analysis of the oral cultures have revealed the molecular epidemiology of the strains and its associated incidence of pneumonia and sepsis. Additionally, the strains harbored the plasmid encoded *mcr-2* resistant gene for colistin resistance.^34^ The reason may be due to the *A. baumannii* being resistant to dryness, disinfectants,^35^ and its biofilm formation capacity on the inanimate surfaces fixed to ICU patients.^36^ A high incidence rate of oral colonization in hospitalized patients is evidenced with increased mortality as well.^37^ Studies also show that *A. baumannii* colonization plays a vital role in the rapid spread of these bacterium in both dental and hospital health care settings.

### Possible sources of oral colonization by A. baumannii

*A. baumannii* may enter the oral cavity via various sources, initiating efficient colonization on the oro-mucosal layers. The infections might also be aggravated in hospitalized and/or immune-compromised patients, as well as in those with oral cancers. The following are some of the most common sources of *A. baumannii* listed in various documents.

### Dental materials

Tray set-ups and tools used for dental care are possible sources for the temporary and permanent survival of *A. baumannii*. Investigations on the pathogenicity of *A. baumannii* in patients using thermoplastic retainers have found higher biofilm forming capacity and stronger adhesion to the retainer materials in comparison with other oral bacterium.^38^ Dental implants used for peri-implantitis cases were also known to be contaminated by *A. baumannii* and many *in vitro* decontamination procedures have been applied to eradicate the bacteria with significant results. This suggests that the implant materials may also be a suitable source for the bacterial colonization in dental health care environments.^39^ Studies also highlight that *Acinetobacter sp.* contamination of pumice used in dental laboratories have been documented in some countries. Improper *Acinetobacter sp.* disinfection of pumice may also be considered as one of the sources of transmission. Moreover, predominance of Gram-negative bacteria, which are contaminants from the dental pumice, has been reported in dentures after minor or major laboratory repair processes, especially the species of *A. calcoaceticus* and *A. lowffi*.^40^

### Dental water source

*A. baumannii* is isolated and identified from various hospital environments on routine microbiological surveillances. In these scenarios, outbreaks are possible in dental health care settings. Recently, a similar outbreak occurred in Tokai University emergency unit, where the tap water was detected as the prominent source of *A. baumannii* infection into the oral cavity when the water was used for dental care. Surprisingly, the strain showed a resistant trait to amikacin and ciprofloxacin.^41^ It is thus suggested that periodical monitoring for the existence of *A. baumannii* in various sources of a dental hospital is essential to successfully control the outbreaks of oral diseases.

### Dentures and denture acrylics

Biofilm analysis on dentures showed that the presence of *A. baumannii* and denture materials acts as reservoirs in harboring the organisms in the oral cavity, further associating with oral infections.^42^ Moreover, the secretion system and the efflux pumps plays a vital role in the removal of antibiotics and other disinfectants, making the organisms stay for a longer time on dentures.^43,44^ Additionally, with the dispersal method, there is the risk that biofilm may detach and progress to cross infections and complicated re- and auto-infections.^45^

### Toothbrushes and brushing techniques

Tooth brushing is considered the best technique to maintain good oral health. Different types of toothbrushes and effective methods of brushing have been periodically taught for the general public. However, the microbial diversity on toothbrushes is yet unclear. A recent study from China documented the results of the toothbrush analyzed via high throughput sequencing from nearly 976 toothbrushes. Interestingly, the study showed the presence of *A. baumannii* with a concluding statement of its survival in the oral cavity further leading to other infectious and systemic infections. In another study analyzing toothbrush on mechanically ventilated patients, nearly 18% of the cases showed the presence of *A. baumannii* with multi-drug resistant properties.^46^ These studies substantiate that toothbrushes may be one of the potential sources of transmission of *A. baumannii* into the oral cavity suggesting the need for the toothbrush care in patients with ventilator associated pneumonia.

### Drug resistant traits of A. baumannii from the oral cavity

Debilitating conditions, such as oral mucositis in the oral cavity, are characterized by erythematous ulcerations of the mucosal layers. Microbiological profiling of the oral lesions has encountered many pathogenic Gram-negative and Gram-positive bacteria. Recently, the oral lesion profiling showed the presence of *A. baumannii* associated with more complicated pseudomembranous lesions on the mucosal layers. Unfortunately, most of the strains were resistant to routine antibiotics of choice exhibiting the property of multi-drug resistance. In the same context, oral colonization by *A. baumannii* among the residents of long-term care facilities (LTCFs) has also been documented with unclear mechanisms of aspiration pneumonia from such subjects.^47^ Most of the strains were resistant to various beta lactam groups of antibiotics mostly mediated by the plasmids. Shockingly, most of the drug resistant strains were high biofilm formers, making the conditions worse in the oral infections. The reason behind this may be due to the modes of horizontal genetic transformations occurring in the biofilms via the process of conjugation, transduction, or transformation, making them more prone to resisting the harsh environmental stresses.^48^

### Correlation/Association of A. baumannii in oral cancers

Oral squamous cell carcinoma, head and neck and oral cancers ranks 6^th^, 7^th^, and 13^th^ among all forms of cancers worldwide, respectively. The treatment strategies highly alter the oral microbiome making the oral cavity a suitable habitat for a wide diverse bacterial profile. In this context, *A. baumannii* was reported with a 4% incident rate following radiation therapy.^49^ In a recent analysis of the oral microbiome associated with gastric carcinogenesis, *A. baumannii* has been documented as one of the oral microbial strains complicating the condition.^50^ A comparative evaluation between the healthy and oral cancer patients showed a significant increase in *A. baumannii* associated with tumorous lesions.^51^ In children with cancer, the swabs from the dorsum of the tongue and mouth showed the presence of *A. baumannii* in association with other oral pathogens.^52^

In cancer patients, reconstructive surgical procedures of the head and neck predispose the entry of *A. baumannii* followed by post-operative surgical infections exhibiting drug resistant traits; it also suggests the need for early recognition of the bacterium in patients with head and neck cancers.^53^ An abundance of *A. baumannii* has also been documented from the more advanced lesions of the oral squamous cell carcinoma.^54^ Iron chelating property is considered as one of the pathogenic mechanisms among *A. baumannii*,^55^ with a recent study documenting the role of iron chelation in the progression of Oral squamous cell carcinoma (OSCC) and the possible interplay with bacterium such as *A. baumannii*.^56^ Microbiome profiles in patients with oral cancer who smoke marijuana also have shown the presence of a diverse group of bacteria along with *A. baumannii*.^57^

### Potential functional association of A. baumannii virulence in OSCC tissues

OSCC tissues and tumor microenvironments harvested for microbiological profiling showed the presence of *Acinetobacter* sp., warranting further studies on its invasion and pathogenesis in tumor tissues.^58^ The various pathological and biochemical changes associated with the depth of the invasion of these bacteria in OSCC cases have documented the overexpression of efflux pumps,^59^ modulation of inflammatory processes,^60^ and biosynthesis of lipopolysaccharides (LPS),^61^ which may attribute to *A. baumannii* invasion in deeper tumor tissues. Assessment on the potential and functional virulence factor associated with *A. baumannii* transcriptional regulator LysR is also evidenced in OSCC tissues. LysR is a type of transcriptional regulator attributing for the virulence of *A.baumannii* and plays a vital role in virulence under OSCC conditions.^62^

Metagenomic analysis of the OSCC tissues and saliva shows the presence of holobionts. At species level, significant presence of *Acinetobacter* has been evidenced from the saliva samples. In the later stages of the OSCC, it is suggested that *Acinetobacter* species aids in the development and further complications in the tumor microenvironment.^63^ The presence of these bacteria in the OSCC tissues is considered the potential indicator in association with the OSCC development. Analysis on the oral microbial profile of the oral cancer patients after radiotherapy has been documented in newer entries of many organisms in a prospective study conducted in a tertiary care center. The oral swabs have revealed 1.4% of *A. baumannii* isolates among the Gram-negative bacteria, being not significantly observed before radiotherapy of the cancer patients. The evidence warrants the early detection and management of *A. baumannii* in the OSCC patients.

## Conclusion

Dental health care workers should be aware of new pathogens like *A. baumannii*, paying special attention to the emergence of the resistant and virulent traits periodically. Proper infection control measures should be strictly implemented in all the dental clinics to curb the sudden outbreaks and further spread of these pathogens. Periodic training of dental professionals and newer educational programs on these pathogens should be developed in every dental care context. These measures are especially advised for high-risk departments, such as oral and maxillofacial surgery, which needs special training programs on early detection of these bacterial sources and compliance with established guidelines. A differential diagnosis on the clinical evaluations incorporating specific detection strategies of these bacteria is also of paramount importance. Continued surveillance, prompt examination and management of these bacteria would warrant long-term patient care rendering a better quality of life for dental patients.
